# Supplementary material for: Semi-Automatic MRI Muscle Volumetry to Diagnose and Monitor Hereditary and Acquired Polyneuropathies
Source: Brain Sci. 2021 Feb 6;11(2):202. doi: 10.3390/brainsci11020202 (PMC7914808; doi:10.3390/brainsci11020202)
Supplement: Supplementary file 1 [file brainsci-11-00202-s001.pdf]

Semi-automatic MRI muscle volumetry to diagnose and monitor hereditary and acquired polyneuropathies

Friederike S. Bähr[1] | Burkhard Gess MD[1] | Madlaine Müller MD[1,2] | Sandro Romanzetti PhD[1] | Michael Gadermayr PhD[3,4] | Christiane Kuhl MD[5] | Sven Nebelung MD[5,6] | Jörg B. Schulz MD[1,7] | Maike F. Dohrn MD[1,8*]

[1] Department of Neurology, Medical Faculty of the RWTH Aachen University, 52074 Aachen, Germany

[2] Department of Neurology, Inselspital Bern, CH-3010 Bern, Switzerland

[3] Institute of Imaging and Computer Vision, RWTH Aachen University, 52074 Aachen, Germany

[4] Salzburg University of Applied Sciences, 5020 Salzburg, Austria

[5] Department of Diagnostic and Interventional Radiology, Medical Faculty of the RWTH Aachen University, 52074 Aachen, Germany

[6] Department of Diagnostic and Interventional Radiology, Medical Faculty, University Düsseldorf, 40225 Düsseldorf, Germany

[7] JARA-BRAIN Institute Molecular Neuroscience and Neuroimaging, Forschungszentrum Jülich GmbH and RWTH Aachen University, 52425 Jülich, Germany

[8] Dr. John T. Macdonald Foundation, Department of Human Genetics and John P. Hussman Institute for Human Genomics, Miller School of Medicine, University of Miami, Miami, FL 33136, USA

*Correspondence: mdohrn@ukaachen.de

Supplementary Material

SUPPLEMENTARY FIGURE 1

Exemplary sequence of the segmentation process via ITK-SNAP.

A. Determination of the region of interest (ROI)

B. Placing the seeds

C. Evolution of the active contour algorithm starting out from the seeds

D. Accomplished segmentation of a thigh

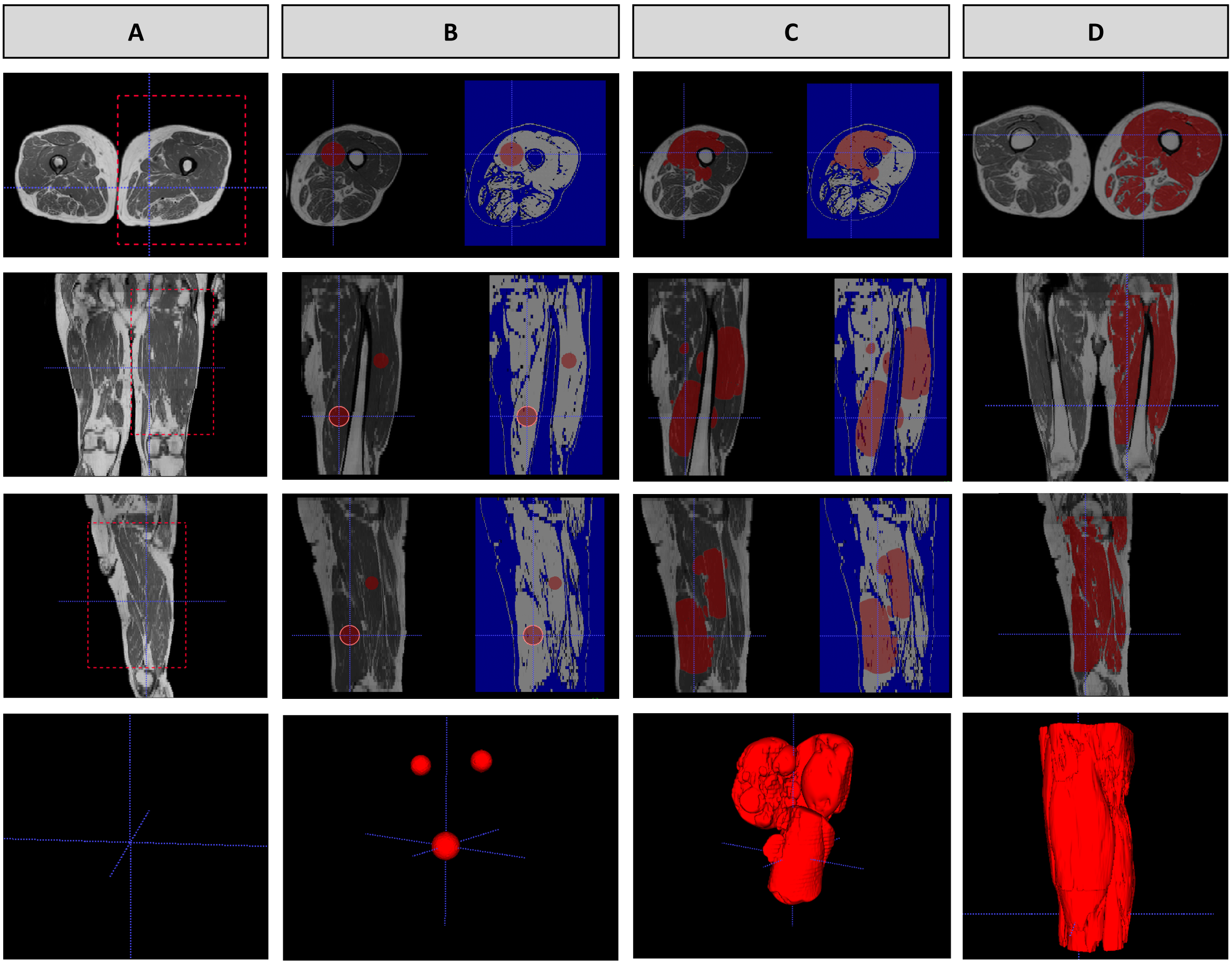

SUPPLEMENTARY TABLE 1

Patient history and clinical examination outcomes in the CIDP and demyelinating CMT cohorts.

Parameters of interest	CIDP			CMT		
	Yes	number*	[%]	Yes	number*	[%]
**First symptom**						
Gait disorder	6	27	22.2	7	12	58.3
Paresthesia	10	27	37.0	0	12	0.0
Numbness	8	27	29.6	0	12	0.0
Pain	11	27	40.7	3	12	25.0
Weakness/ Palsy/ Atrophy	11	27	40.7	1	12	8.3
Muscle cramps	5	27	18.5	0	12	0.0
**Disease progression**						
Stable	17	27	63.0	4	10	40.0
Improving	10	27	37.0	0	10	0.0
Slowly deteriorating	13	27	48.1	5	10	50.0
Rapidly deteriorating	1	27	3.7	2	10	20.0
Fluctuating	4	27	14.8	0	10	0.0
**Current symptoms**						
Neuropathic pain	11	24	45.8	7	10	70.0
Muscle pain	6	23	26.1	6	11	54.5
Paresthesia	19	23	82.6	6	8	75.0
Muscle cramps	14	21	66.7	6	10	60.0
Fine motor skills affected	8	20	40.0	8	12	66.7
Needs a support (walking aid)	7	25	28.0	4	13	30.8
Limited walking distance	16	24	66.7	6	8	75.0
**Examination findings**						
Pes cavus	9	22	40.9	12	13	92.3
Reduced/ Absent patellar reflex	20	25	80.0	8	13	61.5
Reduced/ Absent Achilles tendon reflex	24	25	96.0	10	13	76.9
Steppage gait	12	23	52.2	5	10	50.0
Toe walking affected	14	26	53.8	8	13	61.5
Heel walking affected	19	26	73.1	12	13	92.3
**Sensibility**						
Touch sensation reduced (UL)	3	26	11.5	3	13	23.1
Touch sensation reduced (LL)	14	26	53.8	12	13	92.3
Perception of vibration reduced (UL)	5	25	20.0	3	13	23.1
Perception of vibration reduced (LL)	25	26	96.2	9	13	69.2
Temperature sensation reduced (UL)	7	22	31.8	4	13	30.8
Temperature sensation reduced (LL)	18	22	81.8	12	13	92.3
Pinprick perception reduced (UL)	4	23	17.4	3	12	25.0
Pinprick perception reduced (LL)	17	23	73.9	10	12	83.3
**Receiving IVIG therapy**	22	27	81.5	*Not applicable*		

Note: Percentages and means are rounded to one decimal place.

*(number of included patients concerning the corresponding issue); full cohorts: n (CIDP) = 27, n (demyelinating CMT) = 13

Abbreviations in supplementary table 1: LL, lower limbs; UL, upper limbs; CIDP, chronic inflammatory demyelinating polyneuropathy; CMT, Charcot-Marie-Tooth disease
